# Hybrid Microhydrogels with Sulfonic Groups for the Removal of Methylene Blue from Aqueous Solutions

**DOI:** 10.3390/gels12070613

**Published:** 2026-07-09

**Authors:** Samir E. Esquivel, Yulieth Romero, Víctor H. Campos-Requena, Karla A. Garrido-Miranda, Héctor Aguilar-Bolados, Manuel F. Meléndrez, Julio Sánchez, Daniel A. Palacio

**Affiliations:** 1 Biobased and Bioinspired Biomaterials Research Group and Laboratory of Functional Polymers and Environment, Departamento de Polímeros, Facultad de Ciencias Químicas, Universidad de Concepción, Casilla 160-C, Concepción 4030000, Chile; sesquivel@udec.cl (S.E.E.); yuliethpromero@gmail.com (Y.R.); vcamposr@udec.cl (V.H.C.-R.); 2 Scientific and Technological Bioresource Nucleus (BIOREN-UFRO), Universidad de La Frontera, Temuco 4780000, Chile; karla.garrido@ufrontera.cl; 3 Departamento de Polímeros, Facultad de Ciencias Químicas, Universidad de Concepción, Concepción 3349001, Chile; haguilar@udec.cl; 4 Facultad de Ingeniería, Universidad San Sebastián, Campus Las Tres Pascualas, Lientur 1457, Concepción 4030000, Chile; manuel.melendrez@uss.cl; 5 Departamento de Química Orgánica, Facultad de Química y de Farmacia, Pontificia Universidad Católica de Chile, Santiago 7820436, Chile; julio.sanchez@uc.cl

**Keywords:** microhydrogels, hybrid, alginate, zeolite, sulfonic acids, methylene blue

## Abstract

In this study, sulfonic-functionalized hybrid microhydrogels (MHGs-HF) were developed from alginate via ionic crosslinking by dropwise addition, followed by functionalization at different reaction times (3–12 h). The incorporation of sulfonic groups was confirmed by FTIR and thermal analysis, revealing changes in the thermal stability and residual fraction of the material. The microhydrogels achieved 2000% swelling. Methylene blue (MB) adsorption studies performed using an initial MB concentration of 40 mg L^−1^ showed that MHGs-HF achieved removal efficiencies of up to 80–90% under pH 7.0 and 11.0, while increasing ionic strength significantly reduced MB removal efficiency, particularly in the presence of divalent ions, highlighting the importance of electrostatic interactions in the adsorption process. The experimental results analyzed through the DoE methodology indicated that the optimal conditions for MB removal were 129 ppm MB, an adsorbent dose of 85.5 mg, and a reaction time of 19.7. The kinetic analysis revealed a multistage process, with a rapid initial stage dominated by surface adsorption followed by intraparticle diffusion, reaching equilibrium in 14 h. The experimental data fit the pseudo-first-order model, and the Dubinin–Radushkevich model indicated that the process is governed by physisorption. The equilibrium isotherms were described using the Freundlich model, suggesting a heterogeneous surface with multiple active sites. Taken together, these results position MHGs-HF as versatile and efficient materials for the removal of cationic dyes in complex aqueous media.

## 1. Introduction

Methylene blue (MB), chemically known as 3,7-bis(dimethylamino)phenothiazine, is a thiazine-type dye classified as an organic pollutant. Its complex aromatic structure and cationic nature confer stability, making it difficult to biodegrade and remove using conventional treatment methods [1,2]. Its widespread use spans various industries, from textiles-where it serves as a fiber dye-to pharmaceuticals, where it acts as a sensitizer in the photooxidation of organic compounds. This facilitates the release of the compound into receiving water bodies, food, and soil, exacerbating its environmental impact [3,4]. Its direct discharge into industrial wastewater has been reported at concentrations exceeding 200 mg L^−1^ [5]. Prolonged exposure in humans leads to neurological alterations, gastrointestinal disorders, and even cell death. In aquatic life, it causes oxidative stress, a significant reduction in growth, and reproductive capacity [1,6]. Although various strategies, such as chemical oxidation, membrane separation, and catalytic degradation, have been extensively explored, their implementation on an industrial scale is limited by the generation of toxic byproducts, high energy requirements, and the production of secondary sludge [7,8]. In this context, the adsorption technique emerges as an efficient and versatile alternative, standing out for its simplicity, low operating cost, and the ability to design materials with specific active sites. This process involves the deposition of dye molecules on the surface of an adsorbent material [9]. Notable examples include: activated carbon, conjugated nanomaterials (graphene oxide-based composites and metal–organic frameworks), and clays [10,11,12,13]. However, limitations such as poor reusability and susceptibility to contamination have intensified the development of sustainable bioadsorbents derived from natural polymers in recent years [14].

These materials are highly biocompatible, resistant to fouling, and readily available in nature, making them potential candidates for environmental remediation applications [15]. Among these, alginate stands out as a linear biopolymer matrix with a high anionic charge density. Its structure, composed of β-D-mannuronic acid (M) and α-L-guluronic acid (G) blocks, contains carboxyl (-COOH) and hydroxyl (-OH) functional groups that act as active sites capable of interacting with cationic species through ion exchange, complexation, chelation, electrostatic interactions, and hydrogen bonding mechanisms [16,17]. Furthermore, its hydrophilic nature allows it to be configured into various structures (membranes, fibers, microspheres) and to form hydrogels through cross-linking with divalent cations such as Ca^2+^ or Ba^2+^, making it an ideal scaffold for the incorporation of inorganic phases and targeted chemical modifications [18,19,20,21]. For example, Allangawai et al. synthesized a sodium alginate-based hydrogel for the removal of MB, achieving an adsorption capacity of 51.34 mg g^−1^ under optimal basic conditions, associated with the deprotonation of the polymer’s carboxyl groups [22]. Similarly, Chen et al. prepared sodium alginate/lignin (SA/Lig) hydrogel beads through cross-linking with calcium ions for the removal of methylene blue (MB), exhibiting a maximum adsorption capacity of 254.3 mg g^−1^ and a removal efficiency of 84.8% [23].

When combined with clay minerals or zeolites, alginate-based composites exhibit greater adsorption capacity and improved mechanical strength [24]. Zeolites are microporous crystalline aluminosilicates with a three-dimensional structure composed of tetrahedral AlO_4_^−^ and SiO_4_ units linked by oxygen atoms, which gives them a high surface area and cation exchange capacity [25,26]. Their incorporation into polymer matrices provides new active sites and hierarchical porosity that enhance the degradation of color-forming contaminants [27]. Several studies have demonstrated the potential of these hybrid compounds for MB removal. For example, Nigiz et al. reported efficiencies exceeding 97% using clinoptilolite-alginate (CA) beads [28]. Kazemi and Javanbakht developed a magnetic zeolite/chitosan/alginate bionanocomposite, reporting maximum adsorption capacities of 0.516 and 0.999 mg g^−1^ in batch systems [29]. Abdul Mutalib et al. succeeded in improving adsorption performance through chemical modifications of the zeolite, achieving efficiencies of 100% and a maximum adsorption capacity of 45 mg g^−1^ [30]. For their part, Russo et al. developed a ternary system (alginate/magnetic activated carbon/Y-zeolite), reporting a capacity of 1054 mg g^−1^ and an efficiency of 73% under optimal conditions [31]. Despite these advantages, their performance depends on pH, especially under acidic conditions, where the protonation of the alginate’s carboxylate groups decreases the negative charge density and limits electrostatic interactions with the dye cations, thereby reducing adsorption efficiency. This effect is particularly relevant in alginate-zeolite systems, where MB removal depends largely on these electrostatic interactions [32,33].

Taking advantage of alginate’s inherently favorable chemical composition, one strategy for increasing its adsorption capacity involves functionalizing it with ionizable groups [34]. This modification enables the creation of new active binding sites, an increase in electronegativity, and the stabilization of negative charges in the material [35]. Thus, Metin et al. developed bifunctional magnetic microspheres composed of polyanetol sulfonic acid, alginate, and zeolite, which exhibited high adsorption capacity and a methylene blue removal efficiency of 400 mg g^−1^ and 90%, respectively, attributed to the incorporation of sulfonyl groups into their structure [36]. Recently, Toy et al. developed a sulfonated cellulose adsorbent through reaction with sodium 3-chloro-2-hydroxypropylsulfonate, reporting a maximum adsorption capacity of 156.4 mg g^−1^ and a removal efficiency of over 90% within an operating pH range of 2 to 10 for methylene blue [37].

Despite the promising performance reported for alginate-based hydrogels, alginate–zeolite composites, and sulfonated adsorbents in methylene blue removal, important knowledge gaps remain. Most studies have focused on either the incorporation of inorganic fillers or the introduction of additional functional groups, while the combined effect of both strategies within a single alginate-based microhydrogel system has received limited attention. In particular, the influence of zeolite incorporation together with sulfonic group functionalization on the swelling behavior, adsorption performance, adsorption mechanism, thermodynamic properties, and reusability of alginate microhydrogels has not been systematically investigated. Therefore, the development of hybrid alginate–zeolite microhydrogels functionalized with sulfonic groups represents a promising approach to increase the density of anionic active sites and improve the removal of cationic dyes from aqueous media.

In this context, the objective of this study was to investigate the removal of methylene blue (MB) from aqueous solutions using hybrid alginate-zeolite microhydrogels functionalized with sulfonic groups (SO_3_^−^), which were incorporated to provide active binding sites capable of interacting with the cationic contaminant. The synthesized material was characterized using SEM, FTIR, and TGA; and its adsorption performance was analyzed as a function of pH, ionic strength, contact time, initial dye concentration, and temperature. Finally, kinetic and isothermal models were studied to determine the adsorption mechanism, and its regeneration capacity was evaluated through successive cycles of use.

## 2. Results and Discussion

### 2.1. Spectroscopic Characterization of MHGs-HF

**Figure 1:**
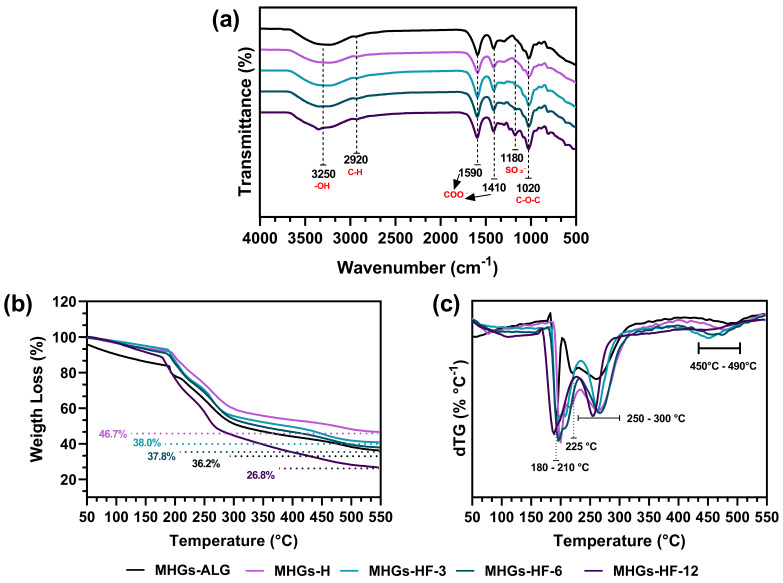
Characterization of MHGs-HF at different reaction times: (**a**) FT-IR, (**b**) TGA and (**c**) dTG.

Figure 1a shows the FT-IR spectra of the MHGs-HF at different reaction times, along with their respective blank samples. The band located around 3250 cm^−1^ is attributed to the stretching vibrations of the -OH group, while the signals observed in the region near 2920 cm^−1^ correspond to the asymmetric and symmetric stretching vibrations of the C-H bond. Likewise, the absorption bands located at 1590 and 1410 cm^−1^ are assigned to the asymmetric and symmetric stretching vibrations of the carboxylate group (COO^−^). The signal at 1020 cm^−1^ is related to the C-O-C stretching vibrations, characteristic of the polysaccharide structure. Subsequently, following chemical modification, a new band appears around 1180 cm^−1^, attributed to the vibrations of the sulfonic group (SO_3_^−^). It is observed that the relative intensity of this signal increases progressively with reaction time, suggesting an increase in the incorporation of sulfonic groups into the alginate structure [38,39].

In addition, Figure 1b,c show the TGA and dTG curves for the different MHGs obtained. In general, the thermal profiles reveal several stages of mass loss associated with dehydration processes, polysaccharide degradation, and transformations of the inorganic species present in the system.

For the MHGs-ALG, three main degradation events were observed at approximately 190, 225, and 260 °C, with a residual mass of 36.2%. Mass loss from room temperature to approximately 200 °C is mainly attributed to the elimination of absorbed water. In the 200–300 °C range, degradation of the alginate main chain occurs, associated with the breaking of glycosidic bonds and the elimination of hydroxyl groups in the form of water. At temperatures above 250 °C, decarboxylation of the polymer occurs, releasing CO_2_ and generating carbonaceous and inorganic calcium-derived residues, such as CaCO_3_, which can subsequently be converted to CaO or Ca(OH)_2_ at higher temperatures [40].

In the case of the MHGs-H, the residual mass increased to 46.7%, which is attributed to the incorporation of the thermally stable inorganic component. Degradation temperatures were observed around 200, 220, and 255 °C, again associated with the decomposition of the alginate matrix. Additionally, a thermal event was identified in the 450–490 °C range, which can be attributed to dehydroxylation processes or structural transformations of the zeolite material, contributing to the increase in the residual fraction [41,42]. Prior to its incorporation into the alginate matrix, the natural zeolite was functionalized with APTMS to introduce amino groups onto its surface. This modification improves the interfacial compatibility between the inorganic filler and the polymeric network through hydrogen-bonding and electrostatic interactions, promoting a more homogeneous dispersion of the zeolite within the microhydrogel structure [43]. As a result, the zeolite can act more effectively as a reinforcing phase, contributing to the enhanced structural stability observed for the hybrid material.

For the MHGs-HF, changes were observed in both the degradation temperatures and the residual mass. In the sample modified for 3 h, thermal events were recorded at 189, 210, and 258 °C, with a residual mass of 38.0%. Similarly, the sample modified for 6 h exhibited degradation at 198, 208, and 255 °C, with a residual mass of 37.8%; finally, in the 12-h sample, a significant decrease in residual mass to 26.8% was observed, with degradation events primarily at 180 and 250 °C. The decrease in residual mass observed in the MHGs-HF is associated with the introduction of sulfonic groups into the material’s structure. During heating, these functional groups promote additional degradation pathways, including their own decomposition with the release of volatile species (SO_2_, SO_3_), as well as the generation of H_2_O and CO_2_ derived from the polysaccharide matrix [44]. Furthermore, the acidic nature of the sulfonic groups can catalyze processes such as dehydration, the breaking of glycosidic bonds, and the decarboxylation of alginate, thereby accelerating the overall degradation of the system [45]. Taken together, these effects lead to greater mass loss and less formation of carbonaceous and inorganic residues at the end of the thermogravimetric analysis.

The particularly low residual mass observed for MHGs-HF-12 may be associated with the higher degree of sulfonic group incorporation achieved after prolonged functionalization. Sulfonic groups can promote additional degradation pathways during heating and act as acidic sites that facilitate dehydration, glycosidic bond cleavage, and decarboxylation reactions within the polysaccharide matrix. As a result, the formation of thermally stable carbonaceous residues is reduced, leading to greater overall mass loss. Furthermore, prolonged functionalization may alter the interactions between alginate chains, calcium ions, and zeolite particles, modifying the degradation behavior of the hybrid network [45,46,47].

### 2.2. Mechanical Characterization of MHGs-HF

The mechanical behavior of the MHGs was evaluated through compression tests, analyzing the stress–strain curves as a function of gap and strain, which revealed the effect of incorporating CHPS on the structural integrity of the system.

**Figure 2:**
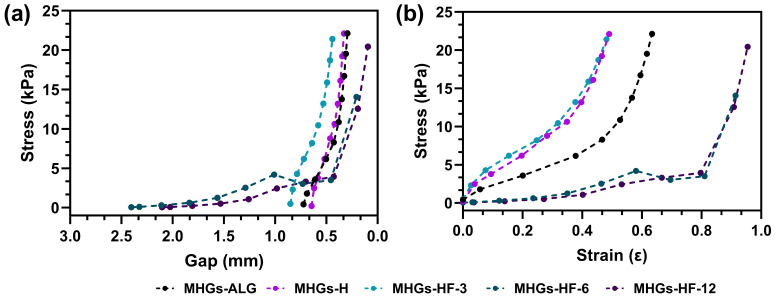
Compressive behavior of MHGs-FH: (**a**) Normal stress as a function of gap and (**b**) strain vs. normal stress.

Figure 2a shows the evolution of normal stress as a function of the gap during compression. In general, all samples exhibited an initial low-stress response, characteristic of highly hydrated polymer networks, where the aqueous phase dominates the initial mechanical response. However, as the gap decreases, clear differences emerge between the systems, highlighting the effect of structural modification on the integrity of the polymer network. The MHGs-ALG, MHGs-H, and MHGs-HF-3 samples exhibit typical mechanical behavior, where stress increases progressively as the gap decreases, reflecting strain hardening associated with network densification. This behavior is attributed to the reduction in free volume, the approach of polymer chains, and the increase in intermolecular interactions under compression [46]. In contrast, the MHGs-HF-6 and MHGs-HF-12 samples exhibited unconventional behavior: as the gap decreased, the stress did not increase proportionally and did not even reach the stress values observed in the other samples. This behavior suggests the occurrence of structural collapse, internal reorganization, or domain-to-domain slippage under severe compression. The higher density of CHPS functionalization likely induces a stiffer network that is less tolerant of large deformations, favoring the occurrence of local failures or loss of mechanical integrity [20,47].

Furthermore, analysis of the stress–strain curves (Figure 2b) reveals changes in the strain capacity of the MHGs depending on their composition and degree of modification. In particular, MHGs-ALG reached a maximum strain close to ε ≈ 0.6 before exhibiting an abrupt increase in stress, indicating the elastic strain limit and the onset of pronounced hardening associated with the extension of the polymer chains. Following the incorporation of zeolite (MHGs-H), this maximum strain decreases to ε ≈ 0.4, a behavior also observed in the sample modified with CHPS for 3 h (MHGs-HF-3). This reduction in deformability suggests an increase in network stiffness, where the presence of inorganic domains and the initial introduction of functional groups restrict the mobility of the chains and limit their ability to reorganize under load [48].

However, this behavior is reversed in the samples with longer modification times (MHGs-HF-6 and MHGs-HF-12), which reach significantly higher strains, in the range of ε ≈ 0.8–1.0, before the abrupt increase in stress. This increase in apparent deformability, far from indicating an improvement in the material’s elasticity, suggests a change in the mechanical response mechanism. In these systems, the high density of CHPS functionalization likely induces a more heterogeneous network, in which rigid regions coexist with less cohesive zones [49]. Under deformation, this structure facilitates internal reorganization processes, slippage between domains, or progressive collapse of the network, allowing for greater deformations before reaching a critical hardening state [50].

### 2.3. Swelling Capacity of MHGs-HF at Different pH

**Figure 3:**
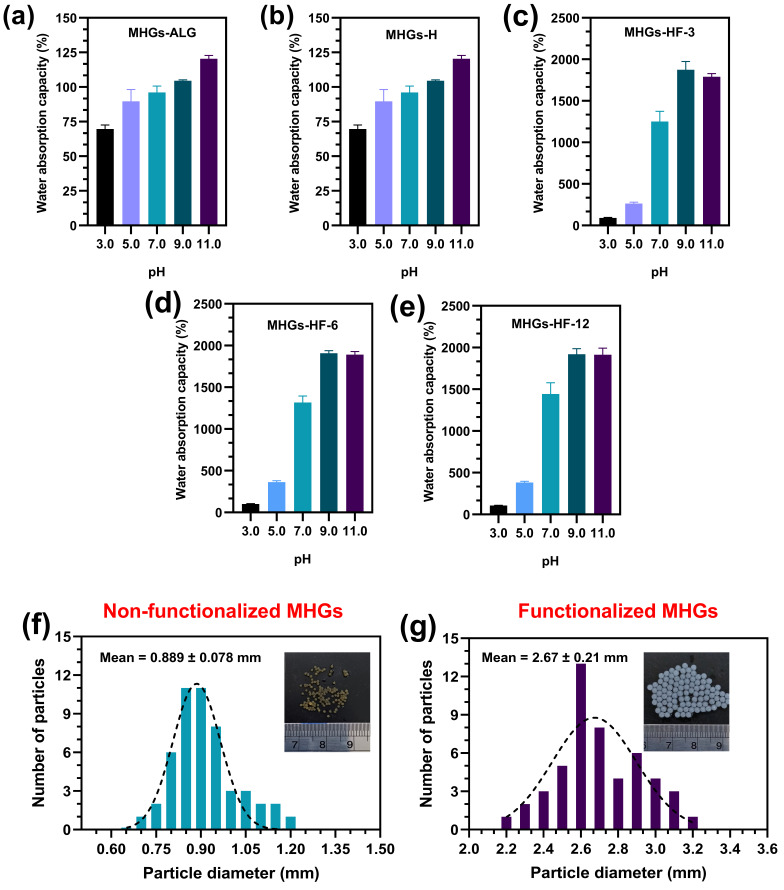
Swelling capacity of MHGs at different pH levels of: (**a**) MHGs-ALG, (**b**) MHGs-H, (**c**) MHGs-HF-3, (**d**) MHGs-HF-6 and (**e**) MHGs-HF-12. Histogram and photos of swollen MHGs: (**f**) unfunctionalized and (**g**) functionalized.

Figure 3 shows the swelling behavior of MHGs-ALG, MHGs-H, MHGs-HF-3, MHGs-HF-6, and MHGs-HF-12 as a function of pH. In general, all materials exhibit a progressive increase in water uptake as pH increases, which is related to changes in the ionization state of the functional groups present in the alginate polymer network. For MHGs-ALG and MHGs-H, water uptake increases moderately from approximately 70% at pH 3.0 to about 120% at pH 11.0. This behavior can be attributed to the gradual deprotonation of the alginate carboxylate groups, which generates electrostatic repulsion between polymer chains and causes the network to expand, facilitating water diffusion and retention within the matrix [51].

In contrast, MHGs-HF exhibit a much more pronounced swelling, exceeding 1800–1900%, under alkaline conditions. This behavior is associated with the presence of sulfonic groups (–SO_3_^−^), which have a high affinity for water and a strong hydration capacity, promoting their swelling [52]. Furthermore, at high pH levels, both the carboxylate groups of alginate (–COO^−^) and the sulfonic groups of CHPS are fully ionized, generating strong electrostatic repulsions between polymer chains [53]. As a result, the three-dimensional network of MHGs expands, increasing the pore volume within the material and allowing for the incorporation of large amounts of water.

On the other hand, Figure 3f,g show the size distribution histograms and representative images of the MHGs swollen in water after 24 h. In the case of MHGs-H (Figure 3f), the average diameter (0.889 ± 0.078 mm) is smaller than that measured for MHGs-ALG (1.23 ± 0.12 mm, Figure S1). This result indicates that the incorporation of the inorganic phase generates a structural reinforcement effect, which limits the expansion of the polymer network and reduces the swelling capacity [54]. In contrast, the functionalized MHG system (Figure 3g) showed a dramatic change, with an average diameter of 2.67 ± 0.21 mm, indicating a significant increase in size following swelling. This behavior is attributed to the presence of sulfonic groups (–SO_3_^−^), which increase water affinity and generate electrostatic repulsions between the polymer chains, thereby promoting the expansion of the three-dimensional network.

**Figure 4:**
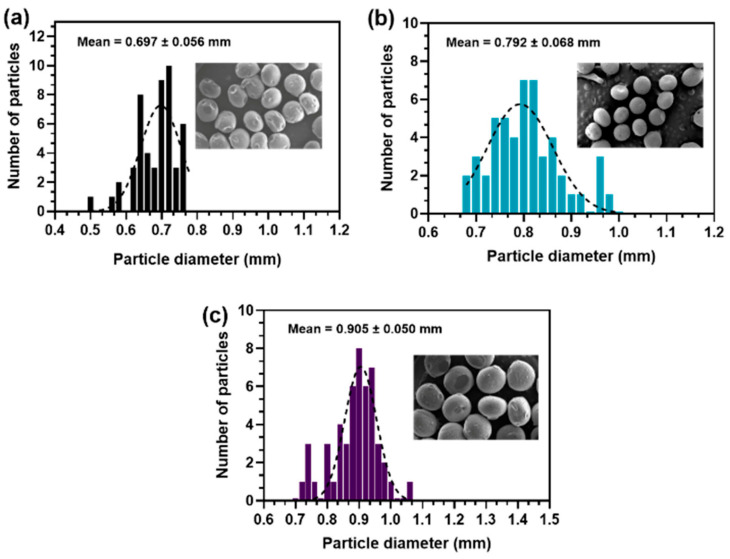
Histograms and SEM images of: (**a**) MHGs-ALG, (**b**) MHGs-H and (**c**) MHGs-HF.

Figure 4 shows the size distribution histograms along with scanning electron microscope (SEM) images of MHGs-ALG, MHGs-H, and MHGs-HF in their dry state. The MHGs-ALG (Figure 4a) have an average diameter of 0.697 ± 0.056 mm and an almost spherical morphology, although they exhibit some surface roughness and slight deformations, which is characteristic of ionically cross-linked alginate systems. For the MHGs-H system (Figure 4b), the average diameter increases slightly to 0.792 ± 0.068 mm, maintaining a relatively controlled distribution. The MHGs exhibit a more defined and uniform shape, suggesting that the zeolite acts as a structuring agent, promoting stability during particle formation. In the case of MHGs-FH (Figure 4c), a significant increase in particle size is observed, reaching an average diameter of 0.905 ± 0.050 mm. SEM images reveal more uniform surfaces, suggesting that the zeolite helps modulate the expansion caused by functionalization by acting as a structural reinforcement. The increase in particle size after CHPS functionalization is consistent with the incorporation of sulfonate moieties into the polymer network. Moreover, the preservation of the spherical morphology after modification indicates that the functionalization process did not compromise the structural integrity of the microhydrogels. The relatively homogeneous surface observed for MHGs-HF suggests a uniform distribution of the modification throughout the material.

### 2.4. Onion D-Optimal Design Analysis

The effect of pH on MB removal was evaluated (Figure S2a,b). Removal efficiency increased from 40–50% at pH 3.0 to 80–85% at pH 7–11, which is attributed to reduced proton competition and the predominance of electrostatic interactions between MB^+^ and deprotonated sulfonic groups (–SO_3_^−^). Considering that MB (*pKa* 3.8) remains mainly in its cationic form above pH 5, adsorption is favored under neutral and alkaline conditions [55,56].

The selection of an Onion D-optimal design instead of a conventional Central Composite Design (CCD) was motivated by the complexity of the experimental domain and the non-uniform distribution of factor levels required in this study. In contrast to CCD, which assumes a regular and symmetric experimental space with evenly spaced factor levels, the Onion D-optimal design allows a more flexible construction of the experimental matrix, particularly when variables are explored over wide and non-linear ranges (e.g., logarithmic scales) or when practical constraints limit the use of standard factorial points. In this work, the independent variables (MB concentration, adsorbent dosage, and contact time) were investigated over broad ranges with multiple intermediate levels (8–9 levels), making a classical CCD inefficient in terms of the number of experiments required and the coverage of the design space. The Onion D-optimal approach enables the combination of factorial, face-centered, and circumscribed layers, resulting in improved space-filling properties and better estimation of quadratic models with a reduced number of experimental runs. Additionally, the D-optimal criterion minimizes the determinant of the covariance matrix of the estimated coefficients, maximizing the statistical efficiency of the model and ensuring robust parameter estimation even under irregular experimental regions. This is particularly advantageous for adsorption systems, where strong non-linear interactions and coupled physicochemical phenomena are expected. Therefore, the Onion D-optimal design was selected to ensure efficient exploration of the experimental space, reduce experimental cost, and improve the predictive capacity of the quadratic model compared to a conventional CCD.

Based on these results, MHGs-HF-3 was selected as the optimal material, as it provides removal efficiencies comparable to longer functionalization times (6 and 12 h), enabling reduced synthesis time without compromising performance. Additionally, although similar removal values (80%) were observed for all materials at pH 7.0, this condition was chosen as the operating pH since it ensures high efficiency without requiring alkaline adjustment.

The pHpzc analysis (Figure S2c) supports these findings, showing values below 5.0 for all materials, indicating that at pH 7.0 the surface is negatively charged, thus favoring electrostatic attraction with MB^+^ [57,58].

Therefore, subsequent experiments were designed using MHGs-HF-3 at pH 7.0, as these conditions maximize removal efficiency while maintaining operational simplicity.

**Figure 5:**
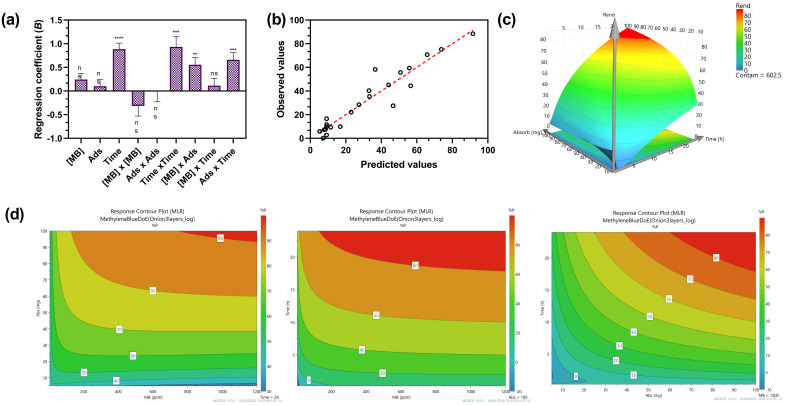
(**a**) Graph of coefficients showing the influence of the variables in the mode (**b**) regression of observed values versus predicted values (**c**) response surface plot of the experimental area under study an (**d**) contour plots showing the effect of MB concentration and adsorbent dosage on MB removal at different fixed contact times (low, medium, and high levels), illustrating the three-factor interaction obtained from the D-optimal design. ** *p* < 0.01, *** *p* < 0.001, and **** *p* < 0.0001; ns: not significant.

The experimental results from the DoE (Table 1) were fitted using MLR, yielding a quadratic model whose polynomial is shown in Equation (1), where *B*_0_ is a constant and the terms *B_n_*, *B_nn_*, and *B_nm_* are the linear, quadratic, and interaction regression coefficients, respectively. The model was validated by an ANOVA test (*p* < 0.001) and showed a regression goodness-of-fit of *R*^2^ = 0.9157 (Figure 5b) and a prediction goodness-of-fit of *Q*^2^ = 0.8497, demonstrating a good correlation between the experimental data and those predicted by the model, as well as confirming its predictive capability within the evaluated experimental range. The RSD value of 9.6990% suggests an acceptable dispersion of the data. The reproducibility value (0.9523) confirms the experimental consistency of the system.

(1) $$Y=B_{0}+B_{1}X_{1}+B_{2}X_{2}+B_{3}X_{3}+B_{11}X_{1}^{2}+B_{22}X_{2}^{2}+B_{33}X_{3}^{2}+B_{12}X_{1}X_{2}+B_{13}X_{1}X_{3}+B_{23}X_{2}X_{3}$$

The model quality was further evaluated using additional statistical parameters. The model validity value (0.556) indicates that the model is acceptable and that there is no significant lack of fit. This is supported by the ANOVA results, where the regression is highly significant (*p* < 0.001) and the lack-of-fit test is not significant (*p* = 0.170), confirming that the model adequately describes the experimental data. The reproducibility of the experimental design was found to be high (0.9523), indicating good agreement among replicate experiments performed at the center points. Although some variability is observed in the replicate runs (Table 1), this dispersion is consistent with the inherent variability of adsorption systems and does not compromise the reliability of the model. Overall, these statistical indicators confirm that the model is robust, predictive, and suitable for describing the response within the studied experimental domain.

Figure 5a shows the regression coefficients associated with the evaluated independent variables. Among them, the quadratic term corresponding to contact time (Time × Time) exhibits the highest statistical significance (*p* < 0.0001), indicating that the adsorption process is strongly governed by a non-linear dependence on contact time. This result is consistent with the kinetic behavior of the system, where the adsorption process initially increases rapidly and then approaches equilibrium, leading to a saturation-type response. Consequently, the quadratic effect of time reflects the transition from the initial adsorption stage to equilibrium conditions.

In contrast, the interaction terms, including [MB] × Time and [MB] × Adsorbent, show lower statistical significance, suggesting that their contribution to the overall response is less dominant compared to the quadratic effect of contact time. Overall, these results indicate that contact time is the most influential factor in the system, primarily through its non-linear behavior, while interaction effects play a secondary role in defining the adsorption efficiency.

Finally, Figure 5c shows the response surface plot for MB removal using MHGs-HF-3 (MB = 602 mg L^–1^), which identifies the optimal amount of adsorbent and contact time to maximize the removal percentage (%R). Increasing the contact time leads to a progressive increase in removal efficiency, reaching maximum values close to 80–90% at prolonged times between 15 and 20 h. However, the response surface shows a clear tendency to plateau under these conditions, suggesting that the system is approaching adsorption equilibrium. This behavior is consistent with the gradual saturation of available active sites. Meanwhile, the variable associated with adsorption capacity also shows a positive effect on efficiency, although not in a strictly linear manner. The curvature observed in the surface indicates the existence of an optimal operating region, where the interaction between the variables maximizes performance. This deviation from linearity confirms that the process cannot be adequately described by a simple first-order relationship, but rather involves coupled physicochemical phenomena. From a mechanistic standpoint, the incorporation of sulfonic groups (–SO_3_^−^) into the polymer matrix favors electrostatic interactions with the cationic dye, promoting adsorption. However, the shape of the response surface suggests that the process is not governed exclusively by surface interactions. Instead, intraparticle diffusion within the polymer network plays a decisive role in controlling the kinetics of the process [59,60].

To further improve the visualization of the three-factor interaction and to better illustrate the combined effect of the variables on MB removal, an additional set of contour plots was generated (Figure 5d). In this representation, the response surfaces are shown as two-dimensional contour maps for fixed values of the third variable at different levels. This approach allows a clearer interpretation of the interaction effects between variables and facilitates the identification of optimal regions within the experimental space. Each subplot represents a different fixed level of the third variable, highlighting how the response evolves as a function of the remaining two factors.

It is important to note that, although quadratic models can predict apparent optima even in systems approaching a plateau, the optimal conditions identified in this study are supported by both the experimental data and the physicochemical behavior of the adsorption system. Specifically, the response surface (Figure 5c) shows a tendency towards saturation at longer contact times and higher adsorbent dosages, consistent with the progressive occupation of active sites and the approach to adsorption equilibrium. The predicted optimum is located within this region of high response, rather than being an isolated mathematical maximum. Furthermore, the experimental runs near the predicted optimum (see Table 1) exhibit removal efficiencies close to the maximum observed values (80–90%), confirming that the model prediction is consistent with the experimental trends. This indicates that the identified optimum reflects a realistic operating region rather than an artifact of the quadratic fitting. Therefore, the model is interpreted as capturing a plateau region with diminishing returns, where the optimum corresponds to the most efficient compromise between variables, rather than a sharp maximum imposed by the polynomial form.

#### 2.4.1. Kinetic Studies of MB Removal

**Figure 6:**
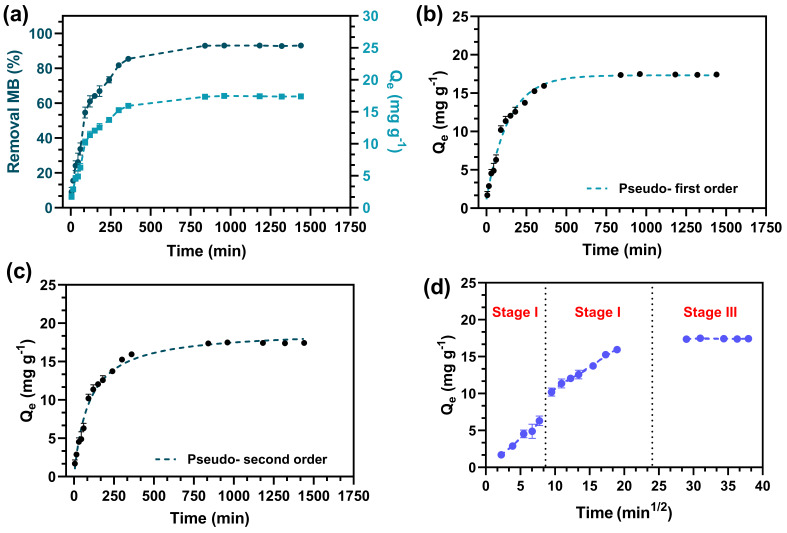
(**a**) Effect of time on MB adsorption by MHGs-HF-, (**b**) the fitting non-linear plots of adsorption kinetic model pseudo-first orde, (**c**) the fitting non-linear plots of adsorption kinetic model pseudo-second order ad (**d**) the fitting linear plots of intra-particle.

Figure 6a shows the evolution of MB removal (%) and adsorption capacity (Q_e_ mg g^−1^) over time for the MHGs-HF-3. It can be seen that both removal efficiency and adsorption capacity increase progressively over time until equilibrium is reached. During the first few minutes of the process, a rapid adsorption stage occurs, where dye removal increases from approximately 9% at 5 min to about 61% at 120 min, while Qe increases from 1.7 to 11.36 mg g^−1^ over the same time interval. This initial rapid stage can be attributed to the high availability of active sites on the surface of the adsorbent and the high concentration gradient between the solution and the material, which favors electrostatic interactions between the cationic MB molecules and the sulfonic groups present in the microhydrogels [61].

Subsequently, between 120 and 360 min, the adsorption rate gradually decreases, reaching removal values close to 85% and an adsorption capacity of approximately 15.95 mg g^−1^. At this stage, the process begins to be controlled primarily by diffusion phenomena, in which MB molecules diffuse into the polymer matrix of the MHGs to occupy the remaining active sites [62]. Finally, the system reaches equilibrium at around 840 min, at which point the removal rate stabilizes at values close to 93%, with a maximum adsorption capacity of approximately 17.4 mg g^−1^, remaining virtually constant until the end of the experiment.

The adsorption kinetics of methylene blue (MB) on MHGs-HF-3 were analyzed using pseudo-first-order, pseudo-second-order, and intraparticle diffusion models in order to understand the mechanisms governing the adsorption process. The parameters obtained from the kinetic fits are presented in Table 2 and plotted in Figure 6b–d.

The pseudo-first-order model provided the best overall description of the experimental kinetic data. In addition to exhibiting a high coefficient of determination (R^2^ = 0.9918), the adsorption capacity calculated by the model, Q*_e_*_(_*_cal_*_)_ = 17.32 ± 0.26 mg g^−1^, was in excellent agreement with the experimental value, Q*_e_*_(_*_exp_*_)_ = 17.42 ± 0.12 mg g^−1^. Therefore, model selection was based not only on the goodness-of-fit parameter but also on the consistency between the predicted and experimental adsorption capacities. The rate constant obtained was *k*_1_ = 7.28 ± 0.49 × 10^−3^ min^−1^, indicating a moderate rate of dye adsorption onto the material. Although the pseudo-second-order model also showed a satisfactory fit (R^2^ = 0.9871), the larger discrepancy between the calculated adsorption capacity, Q*_e_*_(_*_cal_*_)_ = 19.11 ± 0.40 mg g^−1^, and the experimental value suggests a lower predictive accuracy compared to the pseudo-first-order model. Analysis using the intraparticle diffusion model allowed for the evaluation of the possible stages controlling the adsorption process. The results show the presence of multiple linear regions with different intraparticle diffusion constants. In the first stage, *k_id_*_1_ = 0.8057 ± 0.0670 mg g^−1^ min^−1^/^2^ (*R*^2^ = 0.9767) was obtained, corresponding to the rapid initial surface adsorption of the dye onto the active sites of the adsorbent. Subsequently, a second stage with *k_id_*_2_ = 0.6042 ± 0.02 mg g^−1^ min^−1^/^2^ (*R*^2^ = 0.9939) is associated with the gradual diffusion of MB molecules into the interior of the porous matrix of the microhydrogels [63]. The decrease from *k_id_*_1_ to *k_id_*_2_ suggests a transition from rapid surface adsorption and external mass transfer to a slower diffusion process within the swollen microhydrogel network. As the most accessible adsorption sites become occupied, diffusion resistance increases and the apparent diffusion rate decreases.

The kinetic results provide valuable insight into the MB removal mechanism. The excellent agreement between the pseudo-first-order model and the experimental data suggests that the adsorption rate is primarily governed by the availability of accessible adsorption sites and by diffusion-controlled processes rather than by strong chemical bonding. In the present system, MB removal is mainly driven by electrostatic attraction between the cationic dye molecules and the negatively charged sulfonate (–SO_3_^−^) and carboxylate (–COO^−^) groups present in the microhydrogels. In addition, hydrogen bonding interactions and pore diffusion within the swollen polymeric network may contribute to the overall adsorption process. The multi-linear profile observed in the intraparticle diffusion model further confirms that MB removal occurs through a sequence of steps involving external surface adsorption, diffusion through the hydrogel matrix, and final equilibrium saturation of the available adsorption sites. Finally, a third stage with a much gentler slope indicates that the system is approaching equilibrium, where the adsorption rate decreases significantly due to the progressive saturation of the active sites.

#### 2.4.2. MB Adsorption Isotherms at Different Temperatures and Determination of Thermodynamic Parameters

**Figure 7:**
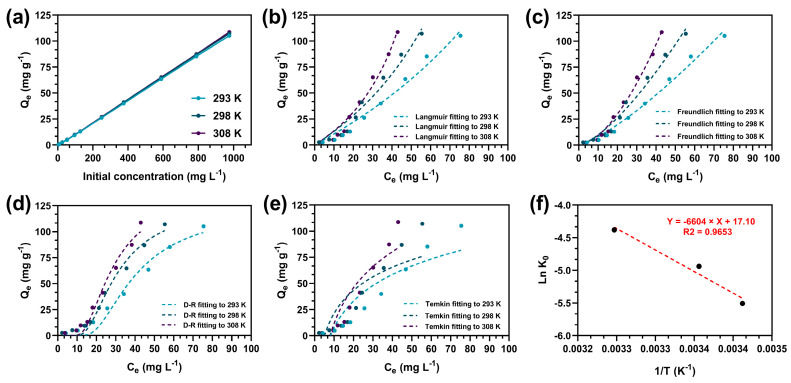
(**a**) Effect of initial MB concentration on MB adsorption by MHGs-HF-3. The non-linear plots f (**b**) Langmui, (**c**) Freundlic, (**d**) D-R, ad (**e**) Temkin, isotherm models ad (**f**) the plot of the thermodynamic model (pH = 7.0, adsorbent dosage: 0.085 g, null ionic strength and contact time: 14 h).

The adsorption of MB onto MHGs-HF-3 was evaluated using the Langmuir, Freundlich, Dubinin–Radushkevich (D–R), and Temkin isotherm models at different temperatures (293, 298, and 308 K), as shown in Figure 7. The parameters obtained from the nonlinear fits are presented in Table 3.

The Langmuir model fit the experimental data well, with coefficients of determination (*R*^2^) ranging from 0.9692 to 0.979. This model assumes monolayer adsorption on a homogeneous surface with a finite number of active sites [64]. The values of the maximum adsorption capacity *Q_e_* ranged from 1.011 to 1.216 mg g^−1^ for the temperatures studied, while the Langmuir affinity constant *K_L_* increased with temperature, rising from 0.00406 L mg^−1^ at 293 K to 0.01254 L mg^−1^ at 308 K. This increase suggests that rising temperature promotes interaction between the dye and the active sites of the adsorbent.

The Freundlich model provided the best overall fit to the experimental data, with *R*^2^ values ranging from 0.9879 to 0.9919, indicating that adsorption occurs on a heterogeneous surface with different adsorption energies. The values of the *K_F_* constant, related to the adsorption capacity, decreased slightly as the temperature increased, while the 1/n parameter showed values greater than 1 across the entire temperature range studied. This behavior suggests cooperative adsorption or complex interactions between the adsorbed MB molecules and the material Surface [64,65].

The Dubinin–Radushkevich model also showed a good *R*^2^ fit ranging from 0.9702 to 0.9843, providing insight into the energy mechanism of the process. The high values of the theoretical adsorption capacity obtained using this model suggest that the material’s porous structure may contribute to the adsorption process [66]. In addition, the average adsorption energy is an important factor to consider when distinguishing between a chemical adsorption process and a physical one, and is given by Equation (2):

(2) $$E=\frac{1}{\sqrt{-2\beta }}$$

where for E < 8 kJ mol^−1^ the physisorption process is dominant over chemisorption, and for values between 8 and 16 kJ mol^−1^ chemisorption predominates. The β values obtained were 0.5079 mol^2^ J^−2^ at 293 K, 0.3011 mol^2^ J^−2^ at 298 K and 0.2511 mol^2^ J^−2^ at 308 K, corresponding to average adsorption energies of approximately 9.92 × 10^−4^, 1.29 × 10^−3^, and 1.41 × 10^−3^ kJ mol^−1^, respectively. These values are less than 8 kJ mol^−1^, indicating that the adsorption of MB onto the material is predominantly governed by physisorption mechanisms [64]. This behavior suggests that the process is primarily controlled by electrostatic interactions between the cationic MB molecules and the negatively charged functional groups present in the modified alginate matrix and the zeolite.

Finally, the Temkin model showed the poorest fit to the experimental data, with R^2^ values of 0.7976, 0.6817, and 0.7698 at 293, 298, and 308 K, respectively. These values indicate that the model provides only a limited description of the MB adsorption process on MHGs-HF-3. Since the Temkin model assumes a linear decrease in the heat of adsorption with increasing surface coverage due to adsorbate–adsorbate interactions, the poor fit suggests that this assumption does not hold in the system studied. This behavior can be attributed to the heterogeneous nature of the adsorbent material, composed of an alginate matrix, zeolite particles, and sulfonate functional groups, which generate multiple types of active sites with different interaction energies.

Figure 7f shows the Van’t Hoff plot, which is used to determine the thermodynamic parameters of the adsorption process. The standard enthalpy values were obtained from the slope and the y-intercept ($\Delta H^{\circ }$) and standard entropy ($\Delta S^{\circ }$), whose results are shown in Table 4. The positive value of $\Delta H^{\circ }=54.9$ kJ mol^−1^ indicates that the adsorption process is endothermic; that is, adsorption is favored by an increase in temperature [67]. Likewise, the positive value of $\Delta S^{\circ }=142.2$ J mol^−1^ K^−1^ s suggests an increase in disorder in the solid-solution interface during the adsorption process, which can be attributed to the release of water molecules previously bound to the adsorbent’s active sites when the dye is adsorbed onto the surface [68,69]. Gibbs energy values (ΔG°) were 13.2, 12.5, and 11.1 kJ mol^−1^ at 293, 298, and 308 K, respectively. Although the positive ΔG° values indicate that the adsorption process is not thermodynamically spontaneous under the evaluated conditions, the progressive decrease in ΔG° with increasing temperature suggests that the energetic barrier to adsorption becomes lower at higher temperatures [70,71]. This behavior is consistent with the positive enthalpy value and confirms that increasing temperature enhances the thermodynamic favorability of the process.

To further assess the performance of the developed material, Table 5 compares the adsorption behavior of MHGs-HF-3 with other alginate-based adsorbents reported in the literature for methylene blue removal. The comparison includes the maximum adsorption capacity, the best-fitting isotherm model, removal efficiency, and desorption/reusability performance when available. This analysis highlights the competitive adsorption performance of the proposed material and provides a broader context for evaluating its potential application in wastewater treatment.

#### 2.4.3. Effect of Monovalent and Divalent Ionic Strength, and Use and Reuse Cycles

**Figure 8:**
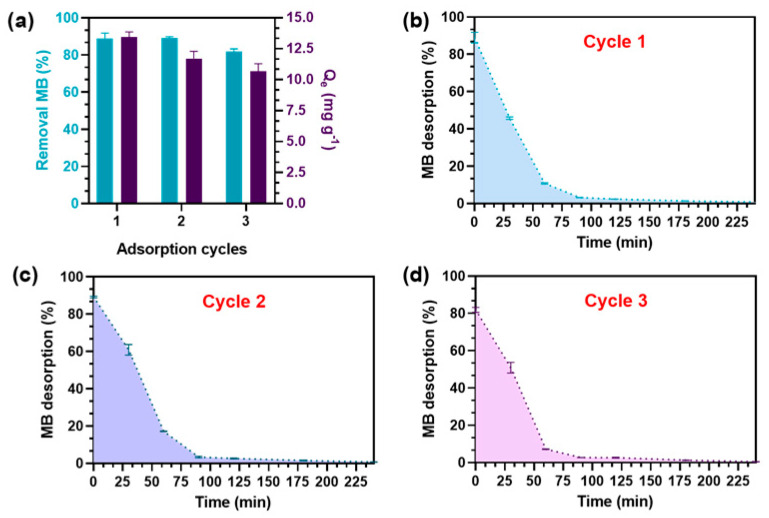
(**a**) Regeneration for the MHGs-HF-3 with MB, and desorption kinetic: (**b**) cycle1 , (**c**) cycle 2 and (**d**) cycle 3.

Figure 8a shows the effect of ionic strength with mono- and divalent salts on MB adsorption. In absence of the salts (control), the system exhibited a removal rate of nearly 90%, with an adsorption capacity (*q_e_*) of approximately 13 mg g^−1^. However, when the ionic strength was increased by adding NaCl, a significant decrease in removal efficiency was observed. At a concentration of 0.2 mol L^−1^, MB removal decreased to approximately 40%, with a *q_e_* of 4.9 mg g^−1^, and continued to decrease progressively until reaching 12% removal and a *q_e_* of 1.7 mg g^−1^ at 1.0 mol L^−1^. A similar, though more pronounced, behavior was observed when CaCl_2_ was used. At 0.2 mol L^−1^, MB removal was approximately 35%, with a *q_e_* of 4.5 mg g^−1^, while at 1.0 mol L^−1^ removal decreased drastically to about 1%, with an adsorption capacity of 0.05 mg g^−1^.

The decrease in adsorption efficiency with increasing ionic strength can be attributed primarily to electrostatic shielding effects and ionic competition for the active sites of the adsorbent. At pH 7.0, the functional groups present in the MHGs, such as carboxylates (-COO^−^) from the alginate and sulfonic groups (–SO_3_^−^) introduced during modification, are deprotonated and generate electrostatic interaction sites favorable for the adsorption of the cationic dye. However, as the concentration of electrolytes in the solution increases, the cations present in the medium compete with the MB molecules for these active sites, reducing the adsorbent–adsorbate interaction. This effect is further enhanced in the presence of Ca^2+^, which can be explained by its higher charge and ability to interact with the functional groups of the alginate. Calcium ions can form ionic bridges with the polymer’s carboxylate groups, decreasing the availability of active sites and reducing the material’s adsorption capacity. Furthermore, the increase in ionic strength reduces the thickness of the electric double layer around the adsorbent, which weakens the electrostatic interactions between the adsorbent and the dye [78,79].

Figure 8b shows the performance of the MHGs-HF-3 during three consecutive cycles of MB adsorption–desorption. In the first cycle, the material exhibits a removal efficiency of nearly 90%. However, a progressive decrease in adsorption efficiency and loading capacity is observed throughout the cycles, reaching approximately 80% in the second cycle and 70% in the third cycle. This loss of performance can be attributed to the partially irreversible adsorption of the dye, as well as to the blocking of high-affinity active sites, which limits the complete regeneration of the material. Additionally, the desorption curves (Figure 8c,d) show a rapid release of the dye during the first 50–60 min, followed by a plateau, indicating the coexistence of adsorption sites with different energies and possible intraparticle diffusion effects. It is important to note that, after the third cycle, the MHGs exhibit a significant loss of structural integrity, manifested in their fracture and fragmentation, which prevents their reuse in additional cycles. This behavior may be associated with mechanical exhaustion of the polymer network, induced by repeated swelling–contraction cycles, as well as with the destabilization of the Ca^2+^-alginate ionic bonds that maintain the structure’s cohesion.

## 3. Conclusions

In this study, sulfonate-functionalized zeolite–alginate hybrid microhydrogels (MHGs-HF) were developed, and their structural, thermal, mechanical, and functional performance was evaluated for the removal of methylene blue (MB) in an aqueous medium. Morphological analysis by SEM revealed an increase in particle size and surface roughness following functionalization, while the incorporation of zeolite favored the formation of more homogeneous and structurally reinforced MHGs. In functional terms, MB removal efficiency increased with pH, reaching values close to 80–90% under neutral and alkaline conditions, consistent with pHpzc values below 5.0, confirming the dominant role of electrostatic interactions. The swelling behavior of the MHGs, particularly at high pH values, was approximately 1800–2000%, attributed to the hydration of ionized groups and electrostatic repulsions between polymer chains. The experimental design showed that the best conditions for MB removal were at 129 mg L^−1^ MB, an adsorbent dose of 85.5 mg, and a time of 19.7 h, with a 95% confidence level. Kinetic studies indicated a multistage adsorption mechanism, with a rapid initial phase dominated by surface interactions, followed by intraparticle diffusion until equilibrium was reached around 14 h. The fit to the pseudo-first-order model and the parameters obtained from the Dubinin–Radushkevich model confirmed a process governed primarily by physisorption. The adsorption isotherms were adequately described by the Freundlich model, highlighting the heterogeneous nature of the material and the coexistence of active sites with different energies. From a thermodynamic standpoint, the process proved to be favorable and endothermic, exhibiting greater affinity at elevated temperatures. Finally, reuse tests showed that, although the material maintains acceptable efficiency over several cycles, structural degradation limits its long-term stability. Future studies should focus on improving the structural stability of the microhydrogels during repeated adsorption–desorption cycles, as well as evaluating their performance in real wastewater matrices containing competing ions and organic contaminants. In addition, the incorporation of alternative crosslinking strategies or reinforcing agents could further enhance the mechanical resistance and reusability of the material, facilitating its potential application in large-scale water treatment processes.

## 4. Materials and Methods

### 4.1. Materials

The sodium alginate used in this study was purchased from (Sigma-Aldrich, Santiago, Chile). The composition of the polysaccharide was determined using the Filipov and Kohn method [80], which allowed us to estimate the ratio of β-D-mannuronate (M) to α-L-guluronate (G) units, as well as the relative proportion of each block in the polymer chain. The results indicated a content of 57% manuronan units and 43% guluronan units, with an M/G ratio of 1.32. Additionally, the molecular weight of the alginate was determined by viscometry and found to be 4.5 × 10^4^ g mol^−1^ (45 kDa) [81]. The zeolite used as a filler in the microhydrogels was a natural zeolite, previously functionalized with 3-aminopropyltrimethoxysilane (APTMS) to introduce amino groups (–NH_2_) onto its surface. Calcium chloride CaCl_2_ (Merck, Providencia, Chile), 95% purity sodium salt hydrate of 3-chloro-2-hydroxy-1-propanesulfonic acid (CHPS), Methylene blue (MB), Sodium chloride (NaCl), sodium hydroxide solution (NaOH) and hydrochloric acid (HCl) at 0.1 mol L^−1^ analytical grade (Merck).

### 4.2. Preparation of Hybrid Microhydrogels Functionalized with Sulfonic Groups (MHGs-HF)

Hybrid microhydrogels were produced by ionotropic gelation through ionic crosslinking of alginate with Ca^2+^ ions, followed by chemical functionalization using sodium 3-chloro-2-hydroxy-1-propanesulfonate (CHPS). To prepare the microhydrogels (MHGs), 1 g of sodium alginate (ALG) was dissolved in 25 mL of deionized water under magnetic stirring at 300 rpm overnight, until a homogeneous 4% (*w*/*v*) alginate solution was obtained. Subsequently, zeolite was added at a mass ratio of 3.5% relative to the alginate, maintaining stirring at 300 rpm until a uniform dispersion of the filler in the polymeric solution was achieved. The resulting alginate-zeolite suspension was transferred via a peristaltic pump, which was set to a flow rate of 1 drop s^−1^. The droplets were allowed to fall from a height of 4 cm into 100 mL of a 2% *w*/*v* CaCl_2_ solution, under constant stirring at 100 rpm, allowing for the instantaneous formation of MHGs via ionic cross-linking. The formed MHGs were recovered and washed with copious amounts of deionized water to remove excess residual CaCl_2_.

The MHGs were then subjected to a chemical modification process by refluxing them in a 5% (*w*/*v*) solution of the CHPS modifier for 3 h at 70 ± 1 °C. The reaction pH was adjusted to 8 by adding 0.02 mol/L NaOH. Once the reaction time was complete, the modified MHGs were subjected to dialysis for 3 days, with water changes every 5 h, to remove residues of the modifying agent. Finally, the MHGs were oven-dried at 35 ± 1 °C for 24 h.

The material obtained under these conditions was designated MHGs-HF-3. The procedure was repeated by increasing the reaction time to 6 h and 12 h to evaluate the effect of modification time on the degree of functionalization. The resulting materials were designated MHGs-HF-6 and MHGs-HF-12, respectively.

Additionally, control MHGs were prepared for comparative purposes: pure alginate (MHGs-ALG), alginate–zeolite (MHGs-H), and CHPS-modified alginate (MHGs-HF). The latter was evaluated using the reaction time that yielded the best results in the methylene blue (MB) removal assays.

### 4.3. Structural and Mechanical Characterization of MHGs-HF

The characterization of the MHGs-HF was conducted using Fourier-transform infrared spectroscopy (FTIR) was conducted using a Jasco 4X spectrophotometer (Tokyo, Japan) equipped with a single-reflection attenuated total reflectance (ATR) accessory fitted with a diamond prism. Spectra were recorded over the range of 4000–400 cm^−1^. Thermogravimetric Analysis (TGA) was carried out using a Netzsch 209 FI IRIS apparatus (Selb, Germany) in a N_2_ atmosphere at a heating rate of 10 °C min^−1^, with a temperature range of 25 to 1000 °C in ceramic capsules. Scanning Electron Microscopy (SEM) was conducted utilizing a JEOL SEM-PROBE CAMECA SU-30 (Gennevilliers, France) model equipped with an EDS instrument. A MCR 702e MultiDrive dynamic mechanical analyzer (Anton Paar, Graz, Austria) was used to perform the compression tests, in compression mode at room temperature. Individual MHGs were placed between the two 10-mm-radius plates and compressed at a rate of 0.1 mm/min, and the change in thickness and the recorded stress were measured. The samples were tested in quintuplicate.

### 4.4. Swelling Capacity

The swelling capacity of the MHGs was determined by placing 30 mg of dry material in 10 mL of water at different pH levels. The samples were stirred at 200 rpm on an orbital shaker for 24 h at room temperature. Subsequently, the hydrated MHGs were removed, excess surface water was removed, and the wet mass was recorded. The degree of swelling was calculated using Equation (3):

(3) $$Swelling\left(\%\right)=\frac{W_{s}-W_{d}}{W_{d}}\times 100\%$$

where $W_{s}$ is the mass of the swollen sample and $W_{d}$ is the initial dry mass.

### 4.5. Determination of the Zero Charge Point (pHpzc)

The zero-charge point (pHpzc) of the material was determined by preparing 0.1 mol L^−1^ NaCl solutions whose initial pH (pHpzc) was adjusted to 3.0, 5.0, 7.0, 9.0, and 11.0 using 0.1 mol L^−1^ HCl or NaOH solutions. Subsequently, 30 mg of MHGs were added to 5 mL of each electrolyte solution, and the suspensions were kept under constant stirring at 200 rpm for 24 h at room temperature. Once the equilibration time had elapsed, the final pH (pH_f_) of each system was recorded. The pHpzc value was determined from the plot of ΔpH versus initial pH (pH_i_), where:

(4) $$\Delta pH=pH_{f}-pH_{i}$$

The pHpzc corresponds to the point at which ΔpH = 0.

### 4.6. MB Removal Tests as a Function of pH

The effect of pH on the removal of methylene blue (MB) was evaluated using 30 mg of adsorbent in 10 mL of a 40 mg L^−1^ MB solution. The initial pH of the solutions was adjusted to 3.0, 5.0, 7.0, 9.0, and 11.0 using diluted HCl or NaOH solutions. The suspensions were kept under constant agitation (200 rpm) in an orbital shaker for 24 h. Subsequently, an aliquot of the supernatant was taken, and the residual MB concentration was determined by UV-Vis spectrophotometry at 664 nm using a UV–Vis spectrophotometer (Thermo Scientific Evolution OnePlus, Sigma-Aldrich, Santiago, Chile). The MB removal percentage (%R) and equilibrium adsorption capacity (Qe) were calculated using Equations (5) and (6), respectively:

(5) $$\%R=\left[\frac{\left(C_{o}-C_{e}\right)}{C_{o}}\right]\times \;100\%$$

(6) $$Q_{e}=\left[\frac{\left(C_{o}-C_{e}\right)}{m}\right]\times \;V$$

where $C_{0}$ is the initial concentration (mg L^−1^), $C_{e}$ equilibrium concentration (mg L^−1^), *V* volume of solution (L) and *m* mass of the microhydrogels (g).

### 4.7. Optimization Through Design of Experiments (DoE)

A three-layer Onion D-optimal experimental design was applied: a first layer consisting of a two-level full factorial design (G-efficiency 100%), a second layer consisting of a three-level face-centered composite design (G-efficiency 100%), and a third layer consisting of a five-level circumscribed composite design (G-efficiency 65.3%). The effect of three independent X-variables on MB removal was evaluated: X_1_ initial MB concentration (mg L^−1^), X_2_ microhydrogel dose (mg), and X_3_ contact time (h) within the ranges shown in Table 6. The range of each variable was studied at 8–9 levels on a logarithmic scale to ensure optimal coverage of the experimental space. The design has a condition number of 5.10, ensuring satisfactory sphericity and orthogonality; for optimization-type designs, this condition number must be <8 [82,83]. The final design consisted of 21 experiments plus 5 center points. The response of interest was the percentage of MB removal (%R). The experiments were conducted in random order to avoid systematic errors. The experimental data were analyzed using multiple linear regression (MLR) to obtain a quadratic model.

Prior to model fitting, the independent variables were transformed using a logarithmic scale to account for the wide range of values explored and to improve model linearity and variance homogeneity. The transformed variables were subsequently standardized and used as inputs (X_1_, X_2_, X_3_) in the multiple linear regression (MLR) model. Therefore, the factors included in Equation 1 correspond to the coded logarithmic values of the original experimental variables (MB concentration, adsorbent dosage, and contact time), rather than their raw values.

#### 4.7.1. Kinetic Studies of MB Adsorption

The kinetic studies were conducted under the optimal conditions determined by the DoE and pH optimization experiments, using an initial MB concentration of 129 mg L^−1^, an adsorbent dosage of 85.5 mg, a solution volume of 10 mL, pH 7.0, and a temperature of 298 K. Contact times ranging from 5 min to 24 h were evaluated. The experimental data were fitted to pseudo-first-order (7), pseudo-second-order (8), and intraparticle diffusion (9) kinetic models:

(7) $$q_{t}=q_{e}(1-e^{-k_{1}t})$$

(8) $$q_{t}=\frac{k_{2}q_{e}^{2}t}{1+k_{2}q_{e}t}$$

(9) $$q_{t}=k_{id}t^{1/2}+C$$

where $q_{t}$ is the amount adsorbed over time t, $k_{1},k_{2}$ are kinetic constants and $k_{id}$ is the intraparticle diffusion constant.

#### 4.7.2. Effect of Ionic Strength

The effect of ionic strength on MB adsorption was evaluated under the optimal conditions of the DoE, using NaCl and CaCl_2_ solutions with concentrations of 0.1, 0.2, 0.5, 0.8, and 1.0 mol L^−1^. The experiments were conducted using the optimal conditions obtained from the experimental design (dose and MB concentration) and the equilibrium time determined from the kinetic studies (14 h). Subsequently, the adsorption capacity (Qe) and removal percentage (%R) were determined using Equations (5) and (6), respectively.

#### 4.7.3. Studies of MB Adsorption Isotherms and Determination of Thermodynamic Parameters

The adsorption isotherms were obtained by varying the initial MB concentration under the optimal experimental conditions determined from the DoE and pH optimization studies. The experiments were carried out at pH 7.0, using 85.5 mg of MHGs-HF-3, a solution volume of 10 mL, and the equilibrium contact time determined from the kinetic studies (14 h). The adsorption experiments were performed at 293, 298, and 308 K. The experimental data were fitted to the Langmuir (10), Freundlich (11), Temkin (12) and Dubinin–Radushkevich (13) models according to the following equations:

(10) $$Q_{e}=\frac{Q_{0}*K_{L}*C_{e}}{1+K_{L}*C_{e}}$$

(11) $$Q_{e}=K_{F}*C_{e}^{\frac{1}{n}}$$

(12) $$Q_{e}=\frac{R*T}{b_{T}}*{ln(A}_{T}*C_{e})$$

(13) $$Q_{e}=Q_{m}exp(-\beta \varepsilon^{2})$$

where *Q_e_* is the amount of adsorbed (mg g^−1^), *C_e_* is the equilibrium concentration of MB (mg L^−1^), *K_L_* is the Langmuir isotherm constant related to the free energy or net enthalpy of adsorption (L mg^−1^), *Q*_0_ is the maximum monolayer adsorption capacity (mg g^−1^), *K_F_* is the Freundlich isotherm constant (mg g^−1^) (L mg^−1^)^1/n^, *n* is a dimensionless parameter indicating the favorability or intensity of adsorption, *A_T_* is the Temkin constant (L g^−1^), *b_T_* is the Temkin constant for heat of sorption (J mol^−1^), *R* is the gas constant (8.314 J mol^−1^ K^−1^) and *T* is the absolute temperature (K), *Q_m_* stands for the maximum adsorption capacity (mg g^−1^), *ε* is the Polanyi potential denoting the work required to remove a molecule to infinity from its location in the adsorption space independent of temperature, and *β* is the Dubinin-Radushkevich isotherm constant, related to the mean adsorption energy.

The thermodynamic parameters of the adsorption process were determined using data obtained from equilibrium isotherms at 293, 298, and 308 K.

Gibbs’ energy change ($\Delta G^{\circ }$) was calculated using (Equation (14)):

(14) $$\Delta G^{\circ }=-RTln\;K$$

Standard enthalpy values ($\Delta H^{\circ }$) and standard entropy ($\Delta S^{\circ }$) were determined using the Van’t Hoff equation (Equation (14)):

(15) $$ln\;K=\frac{-\Delta H^{\circ }}{RT}+\frac{\Delta S^{\circ }}{R}$$

The thermodynamic parameters were obtained from the slope and the y-intercept of the graph of $\ln K_{d}$ vs. 1/T. In this equation, the variable R is 8.314 J mol^−1^ K^−1^, and the variable T is the absolute temperature (K).

#### 4.7.4. Use and Reuse Cycles

To evaluate the reusability of the adsorbent, adsorption–desorption cycles were performed. The adsorption process was carried out using the previously determined optimal conditions (dose, initial MB concentration, and contact time). Subsequently, the loaded microhydrogels were subjected to desorption was performed by immersing the MB-loaded microhydrogels in a 0.5 mol L^−1^ NaCl solution under constant agitation at room temperature. Aliquots were collected at predetermined intervals (30, 60, 90, 120, 180, 240 and 300 min) and the concentration of released MB was determined by UV–Vis spectrophotometry. The desorption process was continued until complete dye release was achieved. After desorption, the microhydrogels were dialyzed against deionized water to remove residual NaCl and MB, dried at 35 ± 1 °C, and subsequently reused in the next adsorption cycle. Desorption efficiency was calculated using Equation (16):

(16) $$Desorption\left(\%\right)=\frac{C_{des}}{C_{abs}}\times 100\%\;$$

where $C_{des}$ is the amount of MB freed and $C_{ads}$ is the amount previously adsorbed. The loading and unloading cycles were repeated to evaluate the stability of the material.

### 4.8. Statistical Analysis

The data are presented as the mean ± standard deviation. One analysis of variance (ANOVA) with Tukey was used to analyze the significant differences between samples. For all tests, the level of significance was set to * *p* < 0.05, ** *p* < 0.01, *** *p* < 0.001, and **** *p* < 0.0001. Graph Prism software version 8.0.2 was used. All experimental design calculations were performed using MODDE 13^®^ software (Sartorius Stedim, Umeå, Sweden).

## Figures and Tables

**Table 1:** Experimental results from the Onion D-optimal experimental design for MB removal.

		*X*-Variables		*Y*-Answers
Exp.	MB (mg L^−1^)	Adsorbent (mg)	Time (h)	%R (%)
1	403.4	36.7	8.2	58.20
2	801.6	68.3	8.2	27.57
3	801.6	36.7	16.1	59.35
4	403.4	68.3	16.1	70.68
5	204.1	20.8	4.2	9.89
6	1000.9	84.2	4.2	40.33
7	1000.9	20.8	20.0	55.68
8	204.1	84.2	20.0	75.24
9	1200	5	0.25	5.84
10	5	100	0.25	7.96
11	1200	100	0.25	5.89
12	5	5	24	35.39
13	1200	5	24	22.16
14	5	100	24	45.14
15	1200	100	24	88.42
16	5	5	1.14	7.29
17	5	13.6	0.25	7.67
18	31.1	5	0.25	28.49
19	1200	22.4	2.45	0.23
20	77.5	100	2.45	9.30
21	77.5	22.4	24	44.34
22	77.5	22.4	2.45	9.94
23	77.5	22.4	2.45	16.50
24	77.5	22.4	2.45	2.76
25	77.5	22.4	2.45	11.64
26	77.5	22.4	2.45	29.92

**Table 2:** Kinetic parameters of MHGs-HF-3 in the removal of MB at 298 K.

Kinetic Models	Parameters	
Pseudo first order	*Q_e_* (*exp*) (mg g^−1^)	17.42 ± 0.12
	*Q_e_* (*cal*) (mg g^−1^)	17.32 ± 0.26
	*K*_1_ (min^−1^)	(7.28 ± 0.49) × 10^−3^
	*R* ^2^	0.9918
Pseudo second order	*Q_e_* (*exp*) (mg g^−1^)	17.42 ± 0.12
	*Q_e_* (*cal*) (mg g^−1^)	19.11 ± 0.40
	*k*_2_ (*g* (mg min)^−1^)	(5.65 ± 0.55) × 10^−4^
	*R* ^2^	0.9871
Intra-particle diffusion	*k_id_*_1_ (mg g^−1^ min^−^½)	0.8057 ± 0.0670
	*R* ^2^	0.9767
	*k_id_*_2_ (mg g^−1^ min^−^½)	0.6042 ± 0.0212
	*R* ^2^	0.9939
	*k_id_*_3_ (mg g^−1^ min^−^½)	17.39 ± 0.2369
	*R* ^2^	0.0052

**Table 3:** MB Adsorption Isotherms for the MHGs-HF-3.

Isotherm Models	Parameters	Temperature		
		293 K	298 K	308 K
Langmuir	*Q_e_* (mg g^−1^)	1.011 ± 0.139	1.216 ± 0.197	1.185 ± 0.106
	*K_L_* (L mg^−1^)	0.00406 ± 0.002	0.00717 ± 0.002	0.01254 ± 0.001
	*R* ^2^	0.9773	0.9692	0.979
Freundlich	*K_F_* (mg g^−1^) (L mg^−1^)^1/^*^n^*	0.392 ± 0.159	0.369 ± 0.189	0.185 ± 0.074
	*1/n*	1.305 ± 0.095	1.423 ± 0.126	1.697 ± 0.107
	*R* ^2^	0.9879	0.9822	0.9919
Dubinin–Radushkevich	*Q_e_* (mg g^−1^)	123 ± 13.14	128.3 ± 8.72	140.9 ± 14.18
	*β* (mol^2^ J^−2^)	0.5079 ± 0.111	0.3011 ± 0.037	0.2511 ± 0.041
	*R* ^2^	0.9702	0.9843	0.9777
Temkin	*A_T_* (L mg^−1^)	36.64 ± 8.42	31.90 ± 10.06	49.59 ± 8.29
	*K_T_* (J mol^−1^)	0.1235 ± 0.067	0.1948 ± 0.401	0.1263 ± 0.031
	*R* ^2^	0.7976	0.6817	0.7698

**Table 4:** Thermodynamic Parameters in the Adsorption of MB by MHGs-HF-3.

∆H° (kJ mol^−1^)	∆S° (J mol^−1^ K^−1^)	∆G° (kJ mol ^−1^)		
		293 K	298 K	308 K
54.9	142.2	13.2	12.5	11.1

**Table 5:** Comparison of alginate-based adsorbents reported for methylene blue removal.

Adsorbent	Best Isotherm Model	Qe (mg g^−1^)	MB Removal (%)	Desorption/Reusability (%)	Reference
Granulos de Algianto con carbon activado-NPs Fe.	Langmuir	14.3	94.3	–	[72]
Sodium alginate/lignin hydrogel beads	--	254.3	84.8	90% after five cycles	[23]
AM/SA/HA/Fe_3_O_4_@Lignite	Freundlich	44.05	96.3	82.14% after five cycles	[73]
alginate/carboxymethyl cellulose-melamine sponge composite (SA/CMC-MeS)	Langmuir	230	99	75% after six cycles	[74]
Sodium alginate and Bentonite	Freundlich	259	98	65% after four cycles	[75]
*Pseudomonas aeruginosa* immobilized within alginate–polyvinyl alcohol (Alg-PVA)	Langmuir	28.9	75	35% after five cycles	[76]
Magnetite–Chitosan–Alginate Nanocomposite (CS/Al@Fe_3_O_4_)	Langmuir	526.3	72	70.8% after four cycles	[77]
MHGs-HF-3	Freundlich	17.5	93	70 after 3 cycles	This work

**Table 6:** Experimental design for the removal of MB.

*X*-Variables	Abbr.	Units	Levels							
*X*_1_ initial concentration of MB	[MB]	(mg L^−1^)	5 (–1)	31.07 (–0.33)	77.46 (0)	204.1 (+0.35)	403.4 (+0.60)	801.6 (+0.85)	1001 (+0.93)	1200 (+1)
*X*_2_ doses of microhydrogels	Ads	(mg)	5 (–1)	20.8 (–0.05)	22.36 (0)	36.7 (+0.33)		68.3 (+0.75)	84.2 (+0.89)	100 (+1)
*X*_3_ contact time	Time	(h)	0.25 (–1)	1.14 (–0.33)	2.45 (0)	4.21 (+0.24)	8.17 (+0.53)	16.1 (+0.83)	20.0 (+0.92)	24 (+1)

## Data Availability

The raw/processed data required to reproduce these findings cannot be shared at this time as the data are also part of an ongoing study.

## Supplementary Materials

The following supporting information can be downloaded at: https://www.mdpi.com/article/10.3390/gels12070613/s1, Figure S1: Histogram and image of MHGs-ALF swollen in water after 24 h; Figure S2: Removal of MB at different pH: (a) MHGs-HF at 3, 6, and 12 h, (b) comparison between the control MHGs and the MHGs-HF-3, and (c) Zero-point load between the control MHGs and the MHGs-HF-3.

## Abbreviations

MHGs	Microhydrogels
MHGs-ALG	Alginate Microhydrogels
MHGs-H	Hybrid Alginate–Zeolite Microhydrogels
MHGs-F	Sulfonic Group-Functionalized Alginate Microhydrogels
MHGs-HF	Hybrid Alginate–Zeolite Microhydrogels Functionalized with Sulfonic Groups
