# Supplementary material for: Hybrid Microhydrogels with Sulfonic Groups for the Removal of Methylene Blue from Aqueous Solutions
Source: Gels. 2026 Jul 9;12(7):613. doi: 10.3390/gels12070613 (PMC13407479; doi:10.3390/gels12070613)
Supplement: Supplementary file 1 [file gels-12-00613-s001.zip › gels-4364663-supplementary.pdf]

Supplementary materials

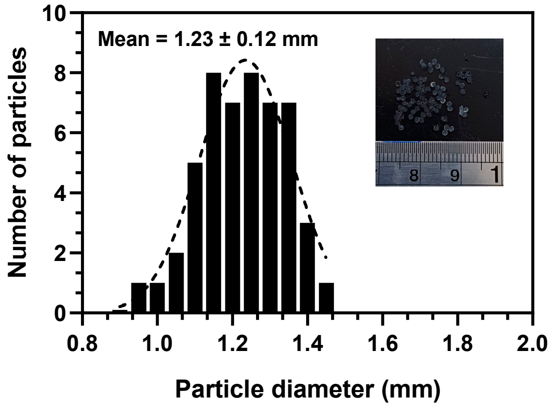

Figure S1. Histogram and image of MHGs-ALF swollen in water after 24 h.

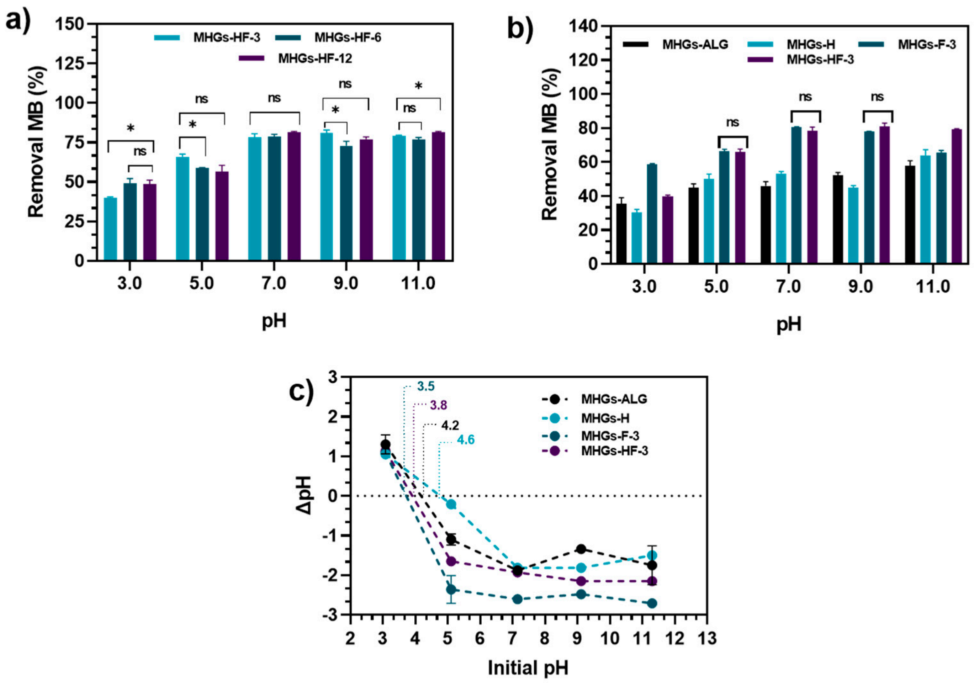

Figure S2. Removal of MB at different pH: (**a**) MHGs-HF at 3, 6, and 12 h, (**b**) comparison between the control MHGs and the MHGs-HF-3, and (**c**) Zero-point load between the control MHGs and the MHGs-HF-3.
